# Integrative Analysis of Epigenome and Transcriptome Data Reveals Aberrantly Methylated Promoters and Enhancers in Hepatocellular Carcinoma

**DOI:** 10.3389/fonc.2021.769390

**Published:** 2021-11-10

**Authors:** Peng Huang, Mengxiang Xu, Haijun Han, Xinyi Zhao, Ming D. Li, Zhongli Yang

**Affiliations:** ^1^ State Key Laboratory for Diagnosis and Treatment of Infectious Diseases, National Clinical Research Center for Infectious Diseases, Collaborative Innovation Center for Diagnosis and Treatment of Infectious Diseases, The First Affiliated Hospital, Zhejiang University School of Medicine, Hangzhou, China; ^2^ Research Center for Air Pollution and Health, Zhejiang University, Hangzhou, China

**Keywords:** epigenetics, DNA methylation, promoter, enhancer, WGBS, RNA-Seq, hepatocellular carcinoma, ChIP-seq

## Abstract

DNA methylation is a key transcription regulator, whose aberration was ubiquitous and important in most cancers including hepatocellular carcinoma (HCC). Whole-genome bisulfite sequencing (WGBS) was conducted for comparison of DNA methylation in tumor and adjacent tissues from 33 HCC patients, accompanying RNA-seq to determine differentially methylated region-associated, differentially expressed genes (DMR-DEGs), which were independently replicated in the TCGA-LIHC cohort and experimentally validated *via* 5-aza-2-deoxycytidine (5-azadC) demethylation. A total of 9,867,700 CpG sites showed significantly differential methylation in HCC. Integrations of mRNA-seq, histone ChIP-seq, and WGBS data identified 611 high-confidence DMR-DEGs. Enrichment analysis demonstrated activation of multiple molecular pathways related to cell cycle and DNA repair, accompanying repression of several critical metabolism pathways such as tyrosine and monocarboxylic acid metabolism. In TCGA-LIHC, we replicated about 53% of identified DMR-DEGs and highlighted the prognostic significance of combinations of methylation and expression of nine DMR-DEGs, which were more efficient prognostic biomarkers than considering either type of data alone. Finally, we validated 22/23 (95.7%) DMR-DEGs in 5-azadC-treated LO2 and/or HepG2 cells. In conclusion, integration of epigenome and transcriptome data depicted activation of multiple pivotal cell cycle-related pathways and repression of several metabolic pathways triggered by aberrant DNA methylation of promoters and enhancers in HCC.

## Introduction

Hepatocellular carcinoma (HCC) is one of the most common malignancies and a growing burden in global health (1, 2), especially in China, which has the highest incidence of HCC due to the high prevalence of hepatitis B virus (HBV) infection (3). Even after decades of research, the 5-year survival rate for liver cancer remains very low, generally less than 5% (4). Therefore, further research on the pathogenesis of HCC and the development of effective diagnosis and prognosis biomarkers is urgently needed.

HCC is a complex disease in which both genetic mutations and epigenetic alterations have been implicated (5). DNA methylation is a critical epigenetic regulator whose aberrations are ubiquitous in many cancers (6). Aberrant DNA methylation has been investigated with several techniques in HCC, including the Human Methylation27 BeadChips (7), Methylation450 BeadChip (5, 8–10), and targeted bisulfite sequencing (11). Alterations in DNA methylation, including both global hypomethylation and regional specific hypermethylation, frequently occur in HCC and related preneoplastic conditions. Hypermethylation events in HCC occur predominantly in promoter-associated CpG islands (CGIs) and correlate with attenuated gene expression (12, 13). Therefore, in HCC, there were recurrent hypermethylated promoter-associated repressions of well-known tumor suppressor genes such as *RASSF1A*, *RUNX3*, *SOCS1*, *HHIP*, *SFRP2*, *APC*, *CDKN1A*, *CDKN2B*, and *CDH* (14–20). However, hypermethylation alterations of CGIs located in the gene bodies of oncogenes were consistently associated with their transcriptional activation (21). Most DNA hypomethylation events in HCC occur in repeat DNA sequences, intergenic regions, and regions outside CGIs (22). Hypomethylation was associated with increased genome instability in HCC (23, 24). Besides, hypomethylation in transcriptional regulatory elements could activate pivotal oncogenic genes like CCAAT/enhancer-binding protein-beta (C/EBPβ) (25). Overall, the transcriptional dysregulations perturbed by abnormal DNA methylation are still not thoroughly clear in HCC.

Furthermore, the commonly used array techniques for studying DNA methylation alterations in HCC lack a good coverage in non-coding regions such as enhancers, which have been implicated as playing pivotal regulatory roles in cancer initiation and development (26, 27). In contrast, whole-genome bisulfite sequencing (WGBS) provides comprehensive single-base-pair resolution-based methylome profiling of more than 90% (>26 million) of all CpGs in the human genome (28). To overcome the limitation of array technologies, WGBS was recently applied to epigenomic profiling of HCC in two studies (24, 25) but with a relatively small sample (generally less than five). Specifically, Dr. Shibata and his colleagues illuminated the interplay between DNA methylation and genetic aberrations by integrating WGBS data and whole-genome shotgun sequencing data (24). On the other side, WGBS was applied to perform global enhancer methylation profiling of three HCC tumors, in which aberrant enhancer hypomethylation of C/EBPβ was discovered and validated as causally linked to C/EBPβ overexpression, thereby contributing to hepatocarcinogenesis through global transcriptional reprogramming (25).

In the present study, we performed WGBS of tumor tissues and paired adjacent tissues from 33 patients for a systematical investigation of the DNA methylation abruption, especially in promoter and enhancer regions, and its associated genes and pathways dysregulated in HCC (**Supplementary Figure S1**). We also aimed to replicate our findings of methylation aberration-associated genes and explore their clinical significances in the TCGA-LIHC cohort to identify potentially effective prognosis biomarkers for HCC.

## Materials and Methods

### Patient Collection

This study was approved by the Institutional Review Board of The First Affiliated Hospital. All tissue samples used in the current study were obtained from patients with HCC who underwent a partial hepatectomy at the First Affiliated Hospital, Zhejiang University School of Medicine. Each specimen was reviewed by a board-certified pathologist to confirm that the frozen section was histologically consistent with tumor or non-tumor tissues. Written informed consent was obtained from each patient.

### WGBS and RNA Sequencing

Paired tumor and adjacent non-tumor tissue samples from 33 HCC patients were subjected to WGBS on the Illumina X Ten platform with the procedures described in our previous WGBS paper (29). Briefly, a 200-ng genomic DNA sample was sheared to about 300-bp fragments by sonication. Then, DNA fragments were subjected to end-repair, addition of adenosine to the 3′ end, and TruSeq adaptor ligation (Illumina, San Diego, CA, USA). Bisulfite conversion was implemented *via* the EZ DNA Methylation kit (Zymo Research, Irvine, CA, USA) according to the manufacturer**’**s protocol. After that, bisulfite-converted DNA was enriched through several cycles of PCR amplification using the KAPA HiFi HotStart uracil DNA polymerases (Kapa Biosystems, Boston, MA, USA). The PCR conditions were set as 45 s at 98°C followed by 10 cycles at 98°C for 15 s, 65°C for 30 s, 72°C for 30 s, ending with 72°C for 1 min. The quality of each WGBS library was assessed by Qubit 2.0 (Life Tech, Carlsbad, CA, USA) and an Agilent 2100 Bioanalyzer. Finally, 150-bp pair-end sequencing was conducted on the Illumina X Ten sequencing platform. High-throughput mRNA-seq was performed for each WGBS sample. Similarly, all 66 RNA samples with high quality (RIN ≥ 7) were applied to the Illumina X Ten platform for sequencing. Specifically, total RNA was extracted and purified using the RNeasy Micro Kit (Qiagen, Valencia, CA, USA) according to the manufacturer**’**s instructions. The quality of RNA was assessed ***via*** an Agilent 2100 Bioanalyzer. Libraries for poly(A)+ RNA were prepared according to the Illumina standard protocol. Constructed libraries were sequenced on HiSeq X Ten platform by WuXi AppTec (Wuxi, Jiangsu China).

### Computational Preprocessing of the Next-Generation Sequencing Data

For raw reads from RNA-seq, Cutadapter (30) (v.1.12) and Trimmomatic (31) (v. 0.33) were applied for adapter removal and trimming of low-quality sequences, followed by FastQC (http://www.bioinformatics.babraham.ac.uk/projects/fastqc) for a quality check. After quality control, clean reads were submitted as the input of Kallisto (32) (v.0.44) for abundance quantification of transcripts based on a gene model download from the GENCODE (v. 29) (33). Normalized expression (transcripts per million [TPM] reads) of each gene was summarized from the transcript level *via* the R package tximport (v. 1.6.0) (34). Non-expressed and low-expressed genes (defined as those with TPM < 0.01 among more than half of the total 66 samples) were excluded from downstream analysis. Clean reads were also aligned against the reference genome by STAR (v. 2.5.2a) (35), and the resulting BAM files were utilized as input for enhancer RNA expression quantification *via* bedtools (v.2.27.1) (36). Besides, the gene count outputs of STAR were used as inputs of DESeq2 (v.1.18.1) (37) for identification of differential expressed genes (FDR < 5% and |Log_2_FoldChange| > 0.5). The clean WGBS reads that passed preprocessing were aligned with the hg38 reference genome using Bismark (v. 0.16.1) (38) with default parameters. The harvested count information from each strand was then combined. As recommended by the R package DSS (v.2.26.0) developer (39), the smoothing approach was adopted for estimation of smoothed methylation level for all 28.9 million CpGs with default parameters.

### Identification of DML and Differentially Methylated Regions

In order to identify overall significant differential methylation between all tumor and non-tumor samples, a combined Baumgartner–Weiβ–Schindler (BWS) test (40) was applied to carry out age-adjusted differentially methylated loci (DML) detection *via* the R package BWStest (v.0.2.2). We divided all the 33 HCC patients into three age groups: **“**young**”** (age < 55 years; n = 10), **“**medium**”** (55 < age ≤ 65; n = 13), and **“**old**”** (age > 65; n = 10). A single BWS test was performed for each age group on every CpG to obtain two individual BWS *p*-values (*p*_left_ and *p*_right_). Afterward, three one-sided *p*-values were combined as statistic T_left_ (or T_right_) = -2* Σlog_10_(*p*_left_ [or *p*_right_]), and a new statistic T was defined as max(T_left_, T_right_) (40). The empirical distribution of the T statistics of combined BWS test was determined by 2.0 × 10^8^ time permutations. At last, an overall empirical *p*-value was estimated as the combined BWS *p*-values for each CpG. CpG with a combined BWS *p*-value < 1.0 × 10^-5^ was identified as DML for subsequent differentially methylated loci (DMR) calling.

Tumor-associated DMRs were determined by R script with the following two steps: 1) DML were combined into pre-DMRs if the distance between neighbor CpGs was < 200 bp; and 2) all CpGs located between the start and the end of each pre-DMR were included as a final DMR. The arithmetic mean of T statistics for all CpGs in each DMR was calculated for estimating the empirical combined BWS *p*-value for each DMR. Group-level methylation was estimated as the arithmetic average of DNA methylation of all CpGs in each corresponding DMR.

### Annotation of DML and DMRs

Annotation of the genomic location of each identified DML and DMR was realized by using the function **“**findOverlaps**”** in the R package GenomicRanges (v.1.30.3) (41). Specifically, location annotations were determined according to overlaps (>1 bp) between the range of each DML/DMR and all known genomic regions. These genomic region annotations were defined on the basis of the gene model downloaded from the GENCODE, which included promoter (upstream 1,500 bp and downstream 500 bp from the TSS of each transcript), exon, intron, 5′-UTR, 3′-UTR, and intergenic region.

### Identification of Promoter- and Enhancer-Like DMRs

Functional annotation was performed for all identified DMRs to search for potentially active promoter- and enhancer-like DMRs. Specifically, liver-active promoter/enhancers were obtained according to candidate regulatory element annotation of eight liver-relevant histone ChIP-seq samples, which included five tissue samples (i.e., two tumor and adjacent samples from two HCC patients and one normal liver sample) from a recent integrative epigenomic HCC study (42) and three liver-relevant samples (i.e., one adult liver tissue, one hepatocyte, and one HepG2 cell sample) from the ENCODE database (43). The regulatory element annotation of these five liver tissue samples was based on 10-state chromHMM annotations, whereas those three ENCODE samples were based on five-state candidate regulatory element annotations. Hence, active liver promoters were composed of regions annotated as **“**activeTSS**”** or **“**activePromoter**”** (refer to regions with both H3K4me3 and H3K27ac peaks) in any of those five tissue samples and **“**promoter-like cRE**”** (refer to regions with both H3K4me3 and DNase peaks) in any of those three ENCODE samples. Similarly, active liver enhancers included regions annotated as **“**activeEnhancer**”** (regions with both H3K4me1 and H3K27ac peaks) in any of those five tissue samples and **“**enhancer-like cRE**”** (regions with both H3K27ac and DNase peaks) in any of those three ENCODE samples. Finally, DMRs that overlapped with at least one active promoter/enhancer in any sample were identified as promoter-like or enhancer-like DMRs. Besides, promoter-like DMRs also included DMRs that were annotated as promoters in the genomic location annotation. For identified promoter/enhancer-like DMRs, their promoter/enhancer-like activity scores were calculated as the number of ChIP-seq liver samples in which they were annotated as promoter/enhancer-like regulatory elements. Moreover, as a supplement of annotated enhancers in the public domain, we estimated enhancer RNA (eRNA) expression, which was reported to be a reliable indicator of enhancer activity (44, 45), for all intergenic DMRs *via* bedtools (36) for identification of potential novel enhancers with active enhancer expression (count of reads ≥ 3 in at least one third of tumor or adjacent HCC samples) among our HCC samples.

### Identification of DMR-DEGs

To investigate the effect of the identified tumor-associated DMRs in transcriptional regulation, we performed integrative genomic analysis by integrating the paired DNA methylomic and transcriptomic data from our original HCC cohort and histone ChIP-seq data from the public domain. To ameliorate potential false-positive findings, we focused only on DMRs (|Δ_methylation_| ≥ 0.15) in candidate active regulatory elements, i.e., active promoters and enhancers in the liver. As mentioned earlier, DMRs that overlapped with active promoters or enhancers were identified as promoter/enhancer-like DMRs. For promoter-like DMRs, nearby genes (TSS ≤ 2 kb away from the DMR start or end site) were tested for correlation between mRNA expression and methylation of DMR. Only differentially expressed genes (FDR < 5% and |Log_2_ FoldChange| > 0.5) with a BH-corrected Spearman correlation *p*-value < 0.05 were designated promoter-like DMR-associated genes.

As for genic enhancer-like DMRs, nearby genes whose distances from DMR were < 100 kb were examined for Spearman correlation, and differentially expressed genes with a BH-corrected correlation *p*-value < 0.05 were identified as enhancer-like DMR-associated genes. Regarding those identified intergenic enhancer-like DMRs, neighbor genes within ± 0.5 Mb of those intergenic enhancer-like DMRs were screened for differentially expressed genes with significant DMR-eRNA–gene triple correlation (simultaneous significant Spearman correlation between DMR methylation and eRNA expression, between eRNA and gene expression, and between DMR methylation and gene expression). Additionally, enhancer-like DMR-associated target genes were ruled out of genes that were also identified as promoter-like DMR-associated genes. Those target genes associated with promoter-like DMRs, genic enhancer-like DMRs, or intergenic enhancer-like DMRs were defined as DMR-DEGs.

### Pathway Enrichment Analysis

Genic- and intergenic enhancer-like DMR-associated genes were first combined as enhancer-like DMR-DEGs, along with promoter-like DMR-DEGs which were then used as inputs for enrichment analysis *via* Metascape (v.3.5) (46).

### Identification of High-Confidence DMR-DEGs

Strict screening procedures were imposed to identify DMR-DEGs with high confidence and a low possibility of false-positive results, which would be more applicable to downstream validation. For promoter-like DMRs and genic enhancer-like DMRs, we conducted another genomic location and functional annotation with a much stricter criterion. Specifically, only DMRs overlapping with at least 80% of a promoter region, an active promoter, or an active enhancer were defined as high-confidence promoter/enhancer-like DMRs. Afterward, all DEGs associated with those high-confidence promoter/enhancer-like DMRs were identified as high-confidence DMR-DEGs. As for intergenic enhancer-like DMRs, only target DEGs that were highly positively co-expressed (Spearman correlation coefficient ρ ≥ 0.7) with corresponding eRNAs passed the screening and were included as high-confidence DMR-DEGs.

### Independent Replication and Clinical Significance of DMR-DEGs in the TCGA-LIHC Cohort

*In silico* replication was conducted for each high-confidence DMR–DEG pair using methylomic and transcriptomic data of the TCGA-LIHC cohort. The clinical phenotypes, DNA methylation, and gene expression dataset of the TCGA-LIHC cohort were downloaded *via* the RTCGA (v.1.22.0) R package (47). The 450 k array methylation data were updated to hg38 from hg19 *via* the UCSC genome liftover tool (genome.ucsc.deu/cgi-bin/hgLiftOver). For each high-confidence DMR–gene pair, the availability of all CpG sites in the DMR and active expression of the corresponding target gene were verified in the LIHC-TCGA. If no CpGs existed or the gene was not expressed, the replication was said to have failed for that DMR–gene pair. Differential DNA methylation, differential gene expression, and the correlation between DNA methylation and gene expression were evaluated *via* Wilcox test and Spearman correlation, respectively. A DMR–gene pair was considered to have been replicated in TCGA-LIHC only when there were significant differential methylation, differential gene expression, and consistent (same sign) significant correlation. About the calculation of replication rates of our identified DMR-DEGs in the TCGA-LIHC, it might be unfair to replicate our promoter/enhancer-like DMR-DEGs directly in the TCGA-LIHC cohort, whose DNA methylation levels were profiled by 450k array, especially for DMRs in enhancers, which were rarely covered by 450k array. Thus, DMR-DEGs that failed to replicate were divided into two groups: Group I, replication failure was because no CpG of the DMR was covered in the 450k array; Group II, at least one CpG was available in the 450k array but still failed to replicate the correlated differential methylation and differential expression. Platform-adjusted replicated rates were calculated as: Count_Replicated_ **/**(Count_Type_ _II failure_ + Count_Replicated_).

In the end, the clinical significance of those high-confidence DMR-DEGs was investigated by way of survival analysis and tumor-stage association analysis. Briefly, the overall survival time (OS) and progression-free survival (PFS) time of TCGA-LIHC samples were retrieved from the integrated TCGA pan-cancer clinical data resource (48). Survival analyses for methylation and gene expression were performed on the basis of the univariate Cox proportional hazards regression model. The Kaplan–Meier method was used to create the survival plots, and the log-rank test was employed to compare the difference between survival curves. The optimal cutoffs of DNA methylation, gene expression, and combination of methylation and expression were determined by minimizing the p-values of log-rank tests. The differences in methylation and gene expression in various tumor stages were compared using ANOVA in R (v.3.5).

### *In Vitro* DNA Methylation-Unmasking Treatment

For expression detection, 5 × 10^5^ L02 or HepG2 cells were seeded in 12-well plates and allowed to reach 80%–90% confluence. Then, freshly prepared 5-aza-2-deoxycytidine (5-azadC; decitabine) solution was added to the medium at a final concentration of 100 μM. Cells were allowed to grow for 72 h at 37°C with 5% CO2 and then harvested for RNA extraction and qRT-PCR quantification. The cDNA was reverse-transcribed using the iScript™ cDNA Synthesis Kit (Bio-Rad, Hercules, CA, USA) according to the manufacturer**’**s protocol. The real-time PCR was conducted with SYBR Premix Ex Taq (TaKaRa, Kyoto, Japan). Glyceraldehyde 3-phosphate dehydrogenase (GAPDH) was used as an internal control for amplification of mRNAs. The comparative C_t_ method was used to calculate the relative mRNA expression. There were three replicates for each experimental condition (2 cell lines × 2 treatment concentrations).

## Results

### Age-Dependent Global Hypomethylation in HCC

**Figure 1:**
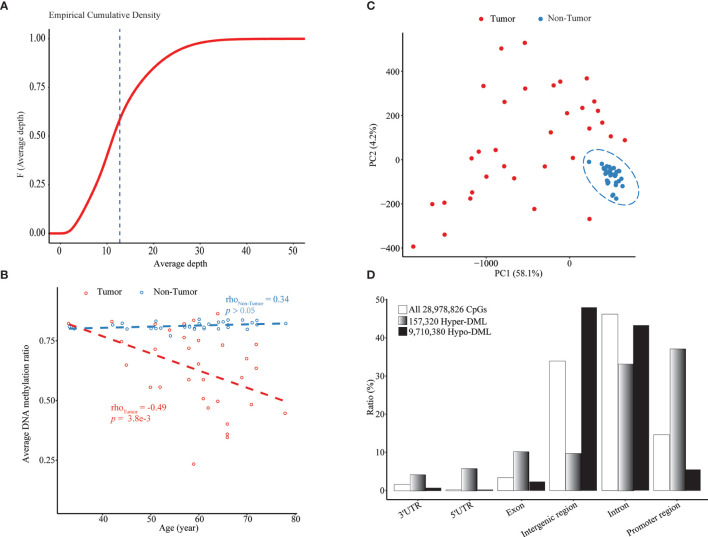
Age-dependent global hypomethylation in HCC. **(A)** Empirical cumulative density plot of average depth of all 28.9 million CpGs profiled by WGBS in all 66 samples. **(B)** Median methylation level of all CpGs in each sample vs. chronological age. **(C)** A PCA plot of all tumor and non-tumor samples based on smoothed methylation levels of all CpGs. **(D)** Genomic location distribution and enrichment of hypomethylated and hypermethylated DML. Upstream 1,500 bp to downstream 500 bp from TSS of each transcript was defined as *“*promoter region*”*.

By WGBS, we obtained the methylation profile of a total of 28,978,826 CpG sites with an average sequencing depth of 12.76 × in 33 pairs of HCC tumor and adjacent non-tumor tissue samples (**Figure 1A**, **Supplementary Figure S2A** and **Supplementary Table S1**). To rescue some CpGs with relatively low depth, smoothed methylation amounts were obtained for all CpGs. We observed a significant methylation difference between tumoral and adjacent tissues and a negative correlation (ρ = -0.49; p = 0.0038) between the average extent of DNA methylation and chronological age in the tumor tissues (**Figure 1B**). This negative correlation also was significant in most genomic regions, including exons, introns, and intergenic regions (**Supplementary Figures S2B–J**). In the PCA plot of all CpGs among all samples, HCCs were clearly separated from paired adjacent tissues, whereas there existed considerable heterogeneities among tumor samples (**Figure 1C**).

Through the combined BWS test, we identified a total of 9,867,700 significant DML between paired HCCs and non-cancerous tissues, including 157,320 hypermethylated DML (hyper-DML) and 9,710,380 hypomethylated DML (hypo-DML). The genomic location annotation of those DML showed that hyper-DML were depleted in the intergenic and intron regions and enriched in other regions, particularly in promoters (defined as -1,500 ~ +500 bp from a TSS) (**Figure 1D**). As for hypo-DML, the pattern was in the opposite direction (**Figure 1D**). Moreover, 47.94% and 43.44% of those hypo-DML were located in the intergenic and intron regions, respectively (**Figure 1D**), which usually were missed in previous arrays or target sequencing-based methylation studies.

### Aberrantly Methylated Promoters and Enhancers in HCC

**Figure 2:**
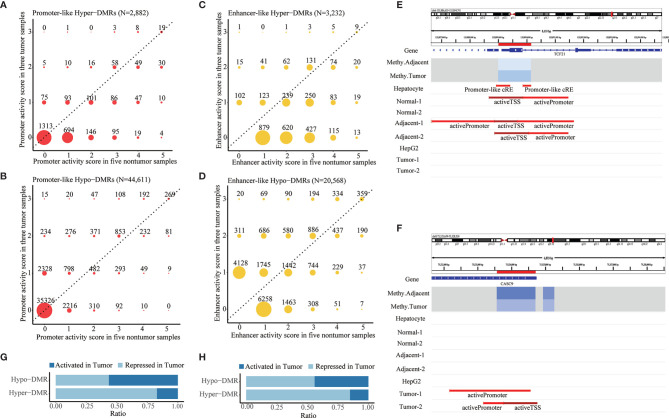
Identification of aberrant DNA methylation in promoter- and enhancer-like regulatory elements. **(A,B)** Distribution of promoter activity of hypermethylated and hypomethylated promoter-like DMRs in tumor *vs*. non-tumor liver ChIP-seq samples. **(C,D)** Distribution of enhancer activity of hypermethylated and hypomethylated enhancer-like DMRs in tumor vs. non-tumor liver ChIP-seq samples. Promoter/enhancer activity scores in tumor and non-tumor reflect counts of tumor and non-tumor liver ChIP-seq samples that show active promoter-/enhancer-related histone peaks in corresponding DMRs. Promoter/enhancers that show greater activity (dots in upper area of dotted line) in tumor condition would be defined as *“*activated (promoter/enhancer) in tumor*”* and vice versa. **(E,F)** Paradigm of repressed and activated promoter associated with differential methylation in HCC. Location of DMRs highlighted in red in the chromosome. *“*Methy.Adjacent*”* and *“*Methy.Tumor*”* refer to average methylation of DMR among adjacent and tumor WGBS samples, respectively. *“*Promoter-Like cRE*”* refers to a genomic region with both H3K4me3 and DNase peak; *“*activePromoter*”* and *“*activeTSS*”* refer to similar regions with both H3K4me3 and H3K27ac peak. **(G,H)** Summarized ratio of *“*activated (promoter/enhancer) in tumor*”* of hypermethylated and hypomethylated promoter/enhancer-like DMRs.

After DMR calling from those DML, we identified 608,279 DMRs composed of 6,924 hypermethylated DMRs (hyper-DMRs) and 601,355 hypomethylated DMRs (hypo-DMRs). Promoter annotation of these DMRs revealed 2,882 promoter-like hyper-DMRs and 44,611 promoter-like hypo-DMRs. Of them, 1,569 promoter-like hyper-DMRs and 9,285 promoter-like hypo-DMRs exhibited active promoter-associated histone peaks (H3K4me3) in at least one of those eight liver-related ChIP-seq samples (**Figures 2A, B** ***)***. From their corresponding enhancer activities in tumor and non-tumor liver ChIP-seq samples, we found 3,232 enhancer-like hyper-DMRs and 20,568 enhancer-like hypo-DMRs (**Figures 2C, D** ***)***. Overall, 61.54% of those 6,924 hyper-DMRs were annotated as promoter or enhancer-like regulatory elements (**Supplementary Figure S3A**), while only 4.17% of those 601,355 hypo-DMRs were annotated as promoter or enhancer-like regulatory elements (**Supplementary Figure S3B**). This indicated that hypermethylation events were much less than hypomethylation events but functionally more critical for transcriptional regulation in HCC.

Those promoter-/enhancer-like DMRs were inferred to be *“*repressed in tumor*”* (**Figure 2E** and **Supplementary Figure S3C**) or *“*activated in tumor*”* (**Figure 2F**; **Supplementary Figure S3D**) according to comparison of their promoter/enhancer activities in tumor and non-tumor samples. In line with the classical negative regulatory relationship between DNA methylation and gene expression, a majority (82.47% and 85.02%, respectively) of those promoter- and enhancer-like hyper-DMRs were recognized as being repressed promoters or enhancers in HCC (**Figures 2G, H** ***)*** whereas about half (44.01% and 56.03%, respectively) of those promoter-like or enhancer-like hypo-DMRs were inferred to be repressed promoters or enhancers in HCC (**Figures 2G, H** ***)***, indicating the potential presence of substantial non-classical positive regulation between DNA methylation and histone modification.

The genomic location annotation for all 6,924 hyper-DMRs and 601,355 hypo-DMRs revealed that there were 641 intergenic hyper-DMRs and 250,932 intergenic hypo-DMRs (**Figure S3E**). Quantification of eRNA expression for all intergenic DMRs revealed 36,651 hypo-DMRs and 309 hyper-DMRs with active eRNA expression in our HCC samples. They were recognized as candidate active enhancers in the liver given that eRNA expression is implicated to be a reliable indicator of enhancer activity. The majority of those intergenic active enhancer candidates appeared to be novel. Specifically, 2,378 of them (6.4%) were annotated as active enhancers in at least one of the eight ChIP-seq liver samples, whereas only 6,622 of all 283,631 intergenic DMRs (2.3%) were annotated as active enhancers, an indication of a nearly three-fold enrichment of known enhancers among our intergenic active enhancer candidates. After correlation analysis between DNA methylation and eRNA expression, 4,833 intergenic hypo-DMRs and 23 intergenic hyper-DMRs exhibited a significant negative methylation-eRNA correlation, whereas 2,126 hypo-DMRs and 52 hyper-DMRs displayed non-classical positive methylation-enhancer regulation(**Figure S3F**). Only these 7,034 intergenic DMRs with both active eRNA expression and significant methylation–eRNA correlation were defined as intergenic enhancer-like DMRs for downstream analyses.

### Genes and Pathways Deregulated by Aberrant DNA Methylation in HCC

**Figure 3:**
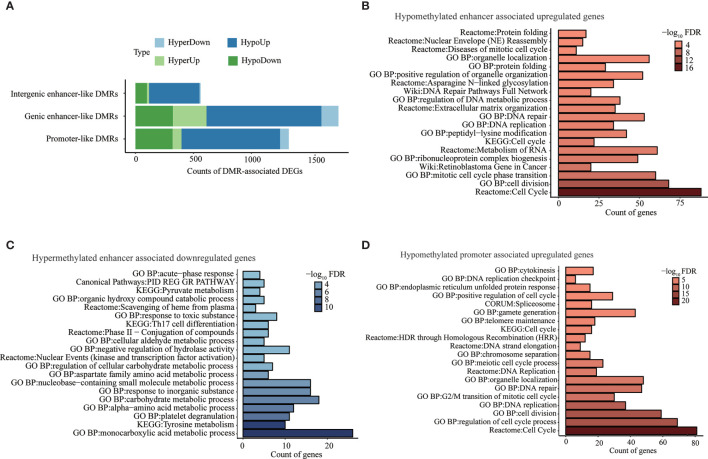
Genes and pathways deregulated by aberrant DNA methylation in promoter- and enhancer-like regulatory elements. **(A)** Counts of genes classically or non-classically associated with identified promoter-, genic enhancer-, and novel intergenic enhancer-like DMRs. “HyperDown” refers to hypermethylated and downregulated gene, “HyperUp” refers to hypermethylated and upregulated gene, “HypoDown” refers to hypomethylated and downregulated gene, and “HypoUp” refers to hypomethylated and upregulated gene. **(B)** Top 20 pathways/process that significantly enriched in activated genes associated with hypomethylated enhancer-like DMRs. **(C)** Top 20 pathways/processes that significantly enriched in repressed genes associated with hypermethylated enhancer-like DMRs. **(D)** Top 20 pathways/processes that significantly enriched in activated genes associated with hypomethylated promoter-like DMRs.

Aberrant methylation associated genes were determined by integrating DNA methylation with transcriptomic data for our identified promoter-like DMRs, genic enhancer-like DMRs, and intergenic enhancer-like DMRs. Specifically, we found a total of 1,323 potential target genes (i.e., promoter-like DMR-DEGs) for all promoter-like DMRs (**Figure 3A** and **Supplementary Table S2.1.1**). As for genic enhancer-like DMRs, we determined 1,751 genes to be their potential targets (i.e., genic enhancer-like DMR-DEGs) (**Figure 3A** and **Supplementary Table S2.1.3**). Regarding intergenic enhancer-like DMRs, there were 562 genes (i.e., intergenic enhancer-like DMR-DEGs) that passed the methylation-eRNA–gene triple correlation examination; i.e., these 562 DMR-DEGs displayed simultaneous significant Spearman correlations between DMR methylation and eRNA expression, between eRNA and gene expression, and between DMR methylation and gene expression (**Figure 3A** and **Supplementary Table S2.1.5**). Overall, 70.0%, 65.0%, and 79.0% of those identified promoter-like DMR-DEGs, genic enhancer-like DMR-DEGs, and intergenic enhancer-like DMR-DEGs appeared to be negatively regulated by DNA methylation.

The pathway enrichment analysis of negatively correlated DMR-DEGs (i.e., HyperDown and HypoUp) demonstrated deregulated DNA methylations in enhancer-induced activation of genes implicated in the cell cycle, retinoblastoma gene in cancer, DNA replication, and DNA repair (**Figure 3B**) accompanied by the repression of genes in various critical metabolism pathways, including monocarboxylic acid metabolic process, tyrosine metabolism, and carbohydrate metabolic process (**Figure 3C**). Similarly, hypomethylated promoters activated genes implicated in the cell cycle, DNA replication, and DNA repair (**Figure 3D**). On the other hand, 75 genes repressed by promoter hypermethylation failed to be enriched in any pathways, possibly because of the small number of genes, but many of them, such as ST8SIA6-AS1 (49) and GRHL2 (50), were reported to play a suppressor role in multiple cancers including HCC (**Table 2**). Pathway enrichment analysis of both negatively and positively methylation-correlated DMR-DEGs displayed similar overrepresented pathways (**Supplementary Table S2.2**).

### *In Silico* Replication and Clinical Significance Investigation of High-Confidence DMR-DEGs

We identified a set of 611 DMR-DEGs with high confidence through strict screening for genes whose associated DMRs overlapped completely with annotated promoters or enhancers from ChIP-seq (**Table 1**). Specifically, we discovered 171 high-confidence promoter-like DMR-DEGs (**Supplementary Table S2.1.2**), 338 high-confidence genic enhancer-like DMR-DEGs (**Supplementary Table S2.1.4**), and 102 high-confidence intergenic enhancer-like DMR-DEGs (**Supplementary Table S2.1.6**). Most of the differential DNA methylation in the promoter and genic enhancer regions (70.18% and 73.96%) exhibited a negative correlation with expression of the target gene, but a considerable proportion (41.18%) of those intergenic enhancers showed hypomethylation-associated gene repression.

**Table 1:** Distribution and replication of 611 high-confident DMR-DEGs.

Type of DMR-DEGs	Count in discovery	Count of type I failure	Count of type II failure	Count passed replication	Replication rate (%)	Adjusted replicationrate (%)
**Promoter-like DMR-DEGs**						
** HyperDown**	12	4	1	7	58.33	87.50
** HypoUp**	108	61	15	32	29.63	68.09
** HyperUp**	21	1	10	10	47.62	50.00
** HypoDown**	30	10	6	14	46.67	70.00
** Total**	171	76	32	63	36.84	66.32
**Genic enhancer-like DMR-DEGs**						
** HyperDown**	14	11	1	2	14.29	66.67
** HypoUp**	236	155	34	47	19.92	58.02
** HyperUp**	34	19	7	8	23.53	53.33
** HypoDown**	54	42	2	10	18.52	83.33
** Total**	338	227	44	67	19.82	60.36
**Intergenic enhancer-like DMR-DEGs**						
** HyperDown**	6	6	0	0	0.00	/
** HypoUp**	54	45	6	3	5.56	33.33
** HyperUp**	0	0	0	0	/	/
** HypoDown**	42	34	2	6	14.29	75.00
** Total**	102	85	8	9	8.82	52.94

HyperDown, hypermethylation-associated downregulated gene; HypoUp, hypomethylation-associated upregulated gene; HyperUp, hypermethylation associated upregulated gene; HypoDown, hypomethylation associated with downregulated gene; Type I replication failure, no CpG available in 450k for corresponding DMR; Type II replication failure, at least one CpG available but no significant differential methylation-associated differential gene expression; replication rate = Count _passed replication_/Count _in discovery_ * 100; Adjusted replication rate = Count _passed replication_/(Count _in discovery_ - Count _type I failure_) * 100.

Literature searching of the identified 56 top differentially expressed high-confidence DMR-DEGs in our HCC sample indicated that 22 of them were implicated in HCC carcinogenesis and the other 15 genes were involved in other types of cancers (**Tables 2**, **3**). Subsequently, 139/661 high-confidence DMR-DEGs were replicated in the TCGA-LIHC cohort, which consisted of 63 promoter-like DMR-DEGs, 67 genic enhancer-like DMR-DEGs, and 9 intergenic enhancer-like DMR-DEGs (**Table 1**). Given the limited availability of WGBS-profiled CpGs in the 450k array, particularly the CpGs in non-coding regions, the raw replication rate of DMR-DEGs was modest except for the promoter-like DMR-associated DEGs. Nevertheless, when the platform effect was adjusted, we achieved a considerably higher replication rate (66.32%, 60.36%, and 52.94%) for the above three groups of DMR-DEGs in the TCGA-LIHC cohort (**Table 1**).

**Table 2:** Top differentially expressed promoter- and genic enhancer-like DMR-DEGs and implicated cancers.

DMR location	Δ_methy_	Gene name	LFC		Dist (bp)	Rho	Rho.padj	TCGA-LIHC replication	Implicated cancer
**Promoter-likeDMR-DEGs**									
**chr8:101492028-101494873**	0.30	GRHL2	-3.36		0	-0.52	9.70E-06	Replicated	HCC (50) and others (51, 52)
**chr14:21022886-21023796**	0.29	TPPP2	-2.93		-313	-0.79	<2.00E-16	Replicated	HCC (53)
**chr6:160122738-160123492**	0.25	SLC22A1	-2.54		949	-0.73	<2.00E-16	Type I failure	Unknown
**chr4:52051223-52052036**	0.26	SPATA18	-2.51		0	-0.56	1.90E-06	Replicated	Breast (54) and others (55, 56)
**chr14:21022886-21023796.1**	0.29	AL161668.4	-2.49		-376	-0.79	<2.00E-16	Type II failure	Unknown
**chr1:58575901-58577030**	0.32	TACSTD2	-2.31		-743	-0.39	0.0012	Replicated	Cholangiocarcinoma (57) and others (58)
**chr8:85465316-85466086**	0.17	CA2	-2.14		1464	-0.44	0.00056	Type I failure	HCC (59) and others (60)
**chr7:128030940-128032690**	0.37	LRRC4	-2.08		0	-0.53	7.20E-06	Replicated	Glioma (61) and Ovarian cancer (62)
**chr6:133889000-133890152**	0.19	TCF21	-1.81		0	-0.39	0.0015	Replicated	HCC (63) and others (64, 65)
**chr1:118983368-118990519**	0.31	TBX15	-1.12		0	-0.4	0.00098	Replicated	Renal cell carcinoma (66)
**chr19:28606496-28607360**	-0.25	AC079466.1	9.26		0	-0.58	6.30E-07	Type I failure	Unknown
**chr7:153409385-153414141**	-0.32	LINC01287	8.83		0	-0.53	7.70E-06	Type I failure	HCC (67) and others (68, 69)
**chr13:64076307-64079199**	-0.35	LINC00355	6.60		263	-0.55	3.00E-06	Type I failure	Gastric (70) and others (71)
**chr10:17386395-17387833**	-0.32	ST8SIA6-AS1	6.23		0	-0.65	3.10E-09	Type I failure	HCC (49) and others (72)
**chrX:133987205-133987743**	-0.24	GPC3	6.20		1310	-0.66	1.30E-09	Type I failure	HCC (73) and others (74)
**chr22:45286491-45287226**	-0.30	UPK3A	5.86		1509	-0.39	0.0012	Type I failure	Unknown
**chr1:26862894-26863264**	-0.26	SFN	5.30		0	-0.69	<2.00E-16	Replicated	HCC (75) and others (76)
**chr15:23565016-23566853**	-0.35	MKRN3	4.40		0	-0.71	1.80E-11	Replicated	Lung cancer (77)
**chr1:43359350-43360396**	-0.16	CDC20	4.18		395	-0.65	1.40E-08	Replicated	HCC (78) and others (79)
**chr13:100088021-100088317**	-0.27	AL355338.1	4.09		-531	-0.42	0.001	Type I failure	Unknown
**Genic enhancer-like DMR-DEGs**									
**chr9:133368447-133369064**	0.22	ADAMTS13	-3.27		-45294	-0.61	1.20E-06	Type I failure	Unknown
**chr1:8004016-8004614**	0.27	AL034417.4	-3.08		12882	-0.5	0.00012	Type I failure	Unknown
**chr7:26365323-26365835**	0.18	AC004540.2	-2.73		-10866	-0.66	7.20E-09	Type I failure	Unknown
**chr19:3428124-3428792**	0.22	SMIM24	-2.47		-51750	-0.41	0.0024	Type I failure	Unknown
**chr5:172871061-172871507**	0.29	DUSP1	-2.15		99866	-0.42	0.0022	Type I failure	HCC (80) and others (81)
**chr11:66717972-66718255**	0.18	SPTBN2	-1.99		-10971	-0.59	1.80E-06	Type I failure	Unknown
**chr1:25686093-25686578**	0.21	MAN1C1	-1.70		68625	-0.39	0.0012	Type I failure	Renal cell carcinoma (82)
**chr16:2036508-2038495**	0.27	SLC9A3R2	-1.26		11152	-0.57	1.40E-05	Replicated	Unknown
**chr2:3535603-3535962**	0.26	ADI1	-1.21		15867	-0.48	5.40E-05	Type I failure	HCC (83) and others (84)
**chr17:4222301-4222630**	0.18	CYB5D2	-1.20		79133	-0.46	0.00011	Replicated	Breast cancer (85) and others (86)
**chr3:124858896-124859415**	-0.15	MUC13	6.72		-94404	-0.52	1.70E-05	Type I failure	HCC (87) and others (88)
**chr5:147773067-147773789**	-0.21	SPINK1	5.45		-57997	-0.45	0.00051	Type I failure	HCC (89) and others (90)
**chr20:43652556-43653516**	-0.30	MYBL2	4.35		-13503	-0.59	1.00E-06	Type I failure	HCC (91) and others (92)
**chr17:44905503-44905963**	-0.21	C1QL1	4.22		-62108	-0.47	0.00081	Type I failure	Lung adenocarcinoma (93)
**chr15:40100406-40101049**	-0.21	BUB1B	4.16		-59974	-0.43	0.0016	Type I failure	Glioblastoma (94) and others (95)
**chr6:44094402-44095522**	-0.19	AL109615.3	4.08		19750	-0.42	0.002	Type II failure	Breast cancer (96)
**chr1:44676848-44677387**	-0.19	KIF2C	3.95		-62431	-0.66	1.10E-08	Type I failure	HCC (97) and others (98)
**chr9:98862188-98863173**	-0.24	COL15A1	3.81		-80006	-0.47	3.00E-04	Type I failure	Unknown
**chr10:5284186-5284850**	-0.18	LINC02561	3.80		12950	-0.64	4.10E-08	Type I failure	Unknown
**chr5:176545906-176546537**	-0.23	GPRIN1	3.23		-63596	-0.59	1.40E-06	Type I failure	Unknown

LFC, estimated log_2_ transformation of fold change of gene expression between tumor and non-tumor (baseline) by DESeq2; Dist, distance between the DMR and associated target genes; Rho, the Spearman correlation coefficient between DNA methylation and gene expression; Rho.padj, BH-adjusted p-value of the Spearman correlation test; Type I failure, no CpG available in 450k for corresponding DMR; Type II failure, at least one CpG available but no significant differential methylation-associated differential gene expression.

**Table 3:** Top differentially expressed intergenic enhancer-like DMR-DEGs and implicated cancers.

DMR location	Δ_methy_	Gene name	LFC	Dist (bp)	Rho.Me	Rho.eG	Rho.MG	TCGA-LIHC replication	Implicated cancer
**chr11:1667908-1668094**	0.23	FAM99A	-2.68	2311	-0.49	0.92	-0.45	Type I failure	HCC (99)
**chr15:74758829-74759080**	0.19	CYP1A2	-4.56	9985	-0.52	0.86	-0.55	Type I failure	HCC (100)
**chr18:31601037-31601741**	0.20	TTR	-2.35	44027	-0.47	0.72	-0.74	Type I failure	Lung cancer (101)
**chr4:154603041-154603623**	0.17	FGG	-1.35	-9344	-0.52	0.72	-0.75	Type I failure	HCC (102) and others (103)
**chr4:154603041-154603623**	0.17	FGB	-1.56	40085	-0.52	0.74	-0.73	Type I failure	Renal cell carcinoma (104)
**chr5:147837794-147838222**	-0.19	SPINK1	5.45	6008	-0.64	0.82	-0.64	Type I failure	HCC (105) and others (90)
**chrX:109740935-109741180**	-0.19	ACSL4	3.76	7532	-0.44	0.7	-0.4	Type I failure	HCC (106) and others (107)
**chr14:19242162-19242285**	-0.23	DUXAP10	3.70	-95445	-0.46	0.83	-0.39	Type I failure	HCC (108) and others (109)
**chr4:49513136-49516430**	-0.27	AC119751.4	3.43	-63403	-0.57	0.72	-0.65	Type II failure	Unknown
**chr18:6683286-6684199**	-0.34	ARHGAP28	2.37	-45519	-0.63	0.71	-0.43	Type I failure	Unknown
**chr8:122448922-122449306**	-0.17	SMILR	2.16	20371	-0.36	0.76	-0.4	Type I failure	Unknown
**chr8:144145816-144146462**	-0.21	TSSK5P	2.15	2152	-0.68	0.73	-0.66	Type I failure	Unknown
**chr1:109309171-109309440**	-0.18	SORT1	2.06	-88511	-0.5	0.74	-0.54	Type I failure	Lung cancer and others (110)
**chr8:122448922-122449306**	-0.17	AC108136.1	1.93	-40509	-0.36	0.71	-0.4	Type I failure	Unknown
**chr8:144347135-144347612**	-0.28	TONSL	1.72	-96832	-0.43	0.71	-0.61	Type I failure	Gastric cancer (111) and others (112)

LFC, estimated log_2_ transformation of fold change of gene expression between tumor and non-tumor (baseline) by DESeq2; Dist, distance between the DMR and associated target gene; Rho.Me, the Spearman correlation coefficient between DNA methylation and enhancer RNA expression; Rho.eG, the Spearman correlation coefficient between enhancer RNA expression and associated target gene expression; Rho.MG, the Spearman correlation coefficient between DNA methylation and associated target gene expression; Type I failure, No CpG available in 450k for corresponding DMR; Type II failure, at least one CpG available but no significant differential methylation-associated differential gene expression.

**Figure 4:**
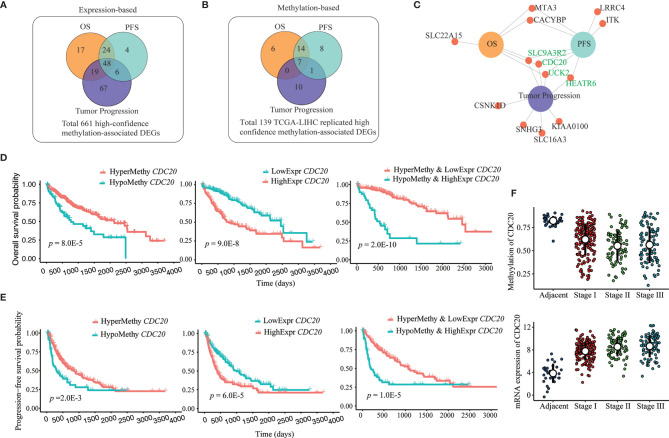
Prognosis and tumor progression-associated biomarkers among DMR-DEGs in HCC. **(A, B)** Overlap between identified survival (OS and PFS) and tumor progression (tumor stage)-associated expression and methylation-based biomarkers among identified 661 high-confidence DMR-DEGs. **(C)** DMR-DEGs, whose expression and methylation were significantly associated with survival and/or tumor progression in HCC. Four genes that associated with both survival and tumor stages are highlighted in green. **(D)** Combination of both promoter hypomethylation and gene upregulation of CDC20 identified more efficient OS biomarkers. **(E)** Combination of both promoter hypomethylation and gene upregulation of CDC20 identified more powerful PFS biomarkers. **(F)** Progressively hypomethylated promoter and increased expression of CDC20 in HCC.

To explore further the clinical significance of our identified 661 potential methylation drivers, we carried out separate survival analyses and tumor stage-association tests based on DNA methylation and transcription data. At the expression level, survival analysis showed that 108 and 82 of those 661 genes were significantly associated with overall survival (OS) and progression-free survival (PFS) (**Figure 4A**), with a high overlap (72 genes) between the two sets of genes. The tumor stage-association test confirmed the significant association between the expression of 140 of the 661 methylation drivers and tumor progression, and 48 of them also were associated with survival (**Figure 4A** and **Supplementary Table S3.1**). As for methylation analyses, most of the 661 high confidence DMR-DEGs, except those 139 DMR-DEGs replicated in the TCGA-LIHC cohort, were excluded because of the low coverage in the 450k methylation array. Among those 139 genes, 27, 30, and 18 were highlighted as OS, PFS, and tumor stage-associated methylation biomarkers (**Figure 4B** and **Supplementary Table S3.2**). In the same manner, there were extensive overlaps among the three sets of clinically relevant genes (**Figure 4B**). Integration of DNA methylation and gene expression data highlighted six OS-associated, eight PFS-associated, and eight tumor stage-associated DMR-DEGs whose transcription and DNA methylation were both plausible biomarkers (**Figure 4C**). Interestingly, five of those six genes whose expression and methylation were both significant OS biomarkers were more powerful (lower log-rank p-values) prognostic biomarkers when considering both expression and methylation data together (**Figure 4D** and **Supplementary Table S3.3**). Similarly, seven of those eight genes whose expression and methylation were both significant PFS biomarkers show higher significance (lower log-rank p-values) when considering both expression and methylation together (**Figure 4E** and **Supplementary Table S3.3**). In addition, four genes (CDC20, UCK2, HEATR6, and SLC9A3R2) were concordantly associated with both survival duration and tumor progression (**Figure 4F** and **Supplementary Tables S3.1, 3.2**).

### Successful *In Vitro* Demethylation Treatment-Based Validation of DMR-DEGs

**Figure 5:**
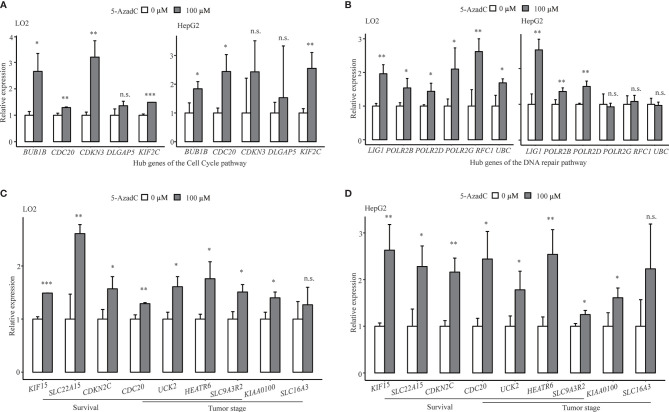
Successful *in vitro* validation of methylation-mediated transcriptional regulation of key pathway hub genes and clinical associated genes. **(A)** Demethylation validation results of five hub genes of cell cycle pathway in LO2 and HepG2 cells. **(B)** Demethylation validation results of selected hub genes from DNA repair pathway in LO2 and HepG2 cells. **(C, D)** Demethylation validation results of selected survival (OS or PFS) and tumor stage-associated genes in LO2 and HepG2 cells. n.s., non-significant; *p < 0.05, **p < 0.01, and ***p < 0.001.

For the sake of validation, 15 top differentially expressed STRING protein–protein interaction (PPI) network hub genes of the overrepresented pathways of DMR-DEGs, including cell cycle, DNA repair, and metabolic pathways, and nine genes significantly associated with OS, PFS, and/or tumor stages were selected for in vitro DNA methylation unmasking validation in LO2 and HepG2 cells. After 5-azadC treatment, 20/23 (87.0%) and 15/23 (65.2%) of those selected genes (one gene, named CDC20, belonged to both the pathway hub gene and the clinically relevant gene) showed significant upregulation in LO2 and HepG2, respectively. For instance, four of the five hub genes in the cell cycle and all six hub genes related to the DNA repair pathway showed apparent upregulation in LO2, and a majority of them also presented upregulation in HepG2 after methylation unmasking (**Figures 5A, B** **)**. Likewise, we obtained similar high validation rates for the remaining pathways (**Supplementary Figure S4**). Furthermore, after 5-azadC treatment in LO2 and HepG2, the qRT-PCR results showed significant upregulation of all seven survival-associated genes and five of the six tumor stage-associated genes (**Figures 5C, D** **)**.

## Discussion

In recent years, large-scale genome-wide DNA methylome studies using methylation array and next-generation sequencing technologies have reshaped our understanding of epigenetic aberrations’ vital roles in tumor formation and maintenance. Owing to the technical merits of WGBS, we identified about 9.8 million differentially methylated CpGs and more than 600,000 regional differential methylations, most of which were located in the intergenic and intronic regions, which could rarely be discovered by the array or target sequencing platform. To the best of our knowledge, this work is the third, but the largest, WGBS-based DNA methylation study in HCC. Considering the small samples of the previous two WGBS studies with sample sizes of eight (five tumor samples and three non-tumor control samples) in one study (24) and of five (two tumor samples and three non-tumor control samples) in the other study (25), they might lack sufficient power to detect methylomic aberrations comprehensively, given the heterogeneity of tumor tissues.

It is well established that HCC is a complex disease contributed to by a disrupted genome that harbors numerous genetic mutations and epigenetic aberrations during the development and maintenance of liver carcinogenesis. Applying an integration of multi-omics data to gain a deeper understanding of the hepatocarcinogenesis mechanisms underlying HCC has become increasingly popular. For instance, the integration of multiple epigenomics data that included DNA methylation, DNA hydromethylation, and four types of histone ChIP-seq data identified novel tumor-suppressor genes for HCC (42). Besides integration with genomic mutations or other epigenomic data, DNA methylomic data most commonly were integrated with genome-wide transcriptome profiling for identification of potential methylation-associated tumor suppressors or oncogenes (113). In our DMR-DEG identification procedure, DNA methylation was systematically integrated with genome-wide gene expression profiling, intergenic eRNA expression, and histone ChIP-seq data. The combination of histone ChIP-seq peak signals and active eRNA expression with DNA methylation profiling contributed to identifying differentially methylated transcription regulatory elements effectively and credibly, followed by integration of gene expression to identify significantly correlated nearby genes as candidate DMR-DEGs for downstream replication and validation. Thus, our strategy would be more powerful and reliable to identify epigenetic drivers with high confidence, in contrast to a simple integrative analysis of the DNA methylome and transcriptome.

Through integrating WGBS-based DNA methylation profiling and RNA-seq based transcriptomic data from paired tumor and adjacent tissues of 33 HCC patients, along with the integration of liver histone ChIP-seq data from the public domain, we identified 661 differential methylated promoter/enhancer-associated target genes and replicated 139 of them in the TCGA-LIHC cohort, which is a high, platform-adjusted, independent replication rate. Moreover, the set of high-confidence DMR-DEGs contains a high proportion of previously experimentally validated HCC driver genes, many other cancer-relevant genes, and some uncharacterized genes with considerable biological function, for instance, cell division cycle 20 (CDC20), a critical coactivator of the cellular division essential complex—anaphase-promoting complex/cyclosome (APC/C), whose overexpression has been associated with the development of a multitude of cancers such as those of the prostate (79) and liver (78). Silencing of CDC20 introduced effective antitumor activity into the orthotopic liver tumor model (114). Although CDC20 is prevalently overexpressed in HCCs (115), the underlying mechanism still was obscure. In our HCC sample, we identified significantly correlated the hypomethylated promoter and transcriptional activation of CDC20, which was replicated successfully in the TCGA-LIHC dataset and validated in methylation-unmasked LO2 and HepG2. Hence, hypomethylated promoter-associated activation might represent a plausible mechanism underlying the widespread dysregulation of CDC20 in HCC. Besides, other known HCC-related genes such as TPPP2 (53), TCF21 (63), GRHL2 (50), and CA2 (59) also were found to be negatively regulated by aberrant promoter DNA methylation in our study. Moreover, the spermatogenesis-associated protein 18 (SPATA18) is a p53-inducible protein involved in the mitochondrial quality-control process, whose dysregulation is associated with cancer. Unlike CDC20, the role of SPATA18 is uncharacterized in HCC, although it also showed concurrent transcriptional repression (115). However, SPATA18 was reported to suppress growth of murine intestinal tumor (116) and human breast cancer (54) *via* mitochondrial quality control. Therefore, our integrative epigenomic analysis might shed new light on the epigenetic-mediated roles of novel genes such as SPATA18 in the process of liver carcinogenesis. Furthermore, accumulating evidence indicates the significance of aberrant enhancer-mediated transcriptional dysregulation in the formation and maintenance of multiple tumors (117, 118), including HCC (25). In the present study, we identified abundant hypomethylated enhancer-associated activated HCC-related genes such as MUC13 (119), SPINK1 (105), and KIF2C (97), plus hypermethylated enhancer-associated repression of known HCC suppressors like DUSP1 (80) and ADI1 (83). Additionally, we found aberrant enhancer-associated dysregulation of genes whose functions are uncharacterized in cancer but harbor possibly essential biological functions. For example, the liver-specific long non-coding (Lnc) gene, FAM99A, was characterized only a few months ago as a powerful regulator of metastasis of HCC (99).

It is well known that DNA methylation modulates gene transcription in negative regulation, especially promoter hypermethylation-induced silencing of tumor suppre sors, which is a hallmark of most cancers. However, there were new studies suggesting that the effect of DNA methylation of CpG islands in gene bodies on transcriptional regulation is different (21, 120). Furthermore, some transcription factors such as CEBPB (121) and RXRA (122) have been reported to prefer methylated CpGs in their binding sites, suggesting a positive correlation between promoter/enhancer methylation and gene transcription. A newly published prostate cancer study also reported extensive, robust associations between DNA hypermethylation and gene upregulation (123), indicating the diversity of epigenetic regulation. In our findings, 181 of those 661 high-confidence DMR-DEGs displayed a positive correlation between methylation and gene expression, and 48 of these 181 genes represented the same non-classical association in the TCGA-LIHC cohort. These 48 genes also contained several noted HCC-relevant genes, such as CELSR3 (124) and PCK1 (125), as well as some genes involved in other cancers, such as NTF3 in breast cancer (126) and TIMD4 in B-cell lymphoma (127).

Our integrative analysis advances the understanding of the disordered methylome of HCC, although there still are several potential limitations. We implemented the first relatively large-scale WGBS-based global DNA methylome profiling of paired tumor and adjacent non-tumor tissues from 33 HCC patients, which covered almost all gene body and intergenic CpG islands that could barely be estimated by the 450k methylation array or target sequencing. However, the average depth of WGBS samples in our study was medium because of the significant cost of WGBS. Besides, we identified a total of 661 high-confidence differentially methylated promoter/enhancer-associated DEGs and achieved a high ratio of successful replication in the TCGA-LIHC cohort after platform limitation adjustment, whereas the considerable DMR-DEGs suffered from lack of replication. Further replication in a larger independent cohort with WGBS-based DNA methylation profiling is greatly needed, which might be a promising method of discovering more epigenetic drivers associated with aberrant methylation in the gene body and intergenic regions. In addition, further validation of methylation-mediated regulation of particular genes *via* technologies like CRISPR, like previous studies (25, 26), were lacking in our present study but would be part of our ongoing works. Besides, it would be better if histone modification, DNA methylation, and gene expression were performed in the same samples, which would provide a more accurate functional annotation of identified DMRs.

Collectively, our integrative analysis of epigenome and transcriptome of HCC convincingly proved the powerful potential of WGBS in uncovering the global DNA methylation aberrations in HCC, especially numerous enhancers in the intron and intergenic regions. Specifically, we identified a group of 661 DMR-DEGs with high confidence, and they were substantially replicated in an independent cohort and validated by *in vitro* methylation unmasking experiments. Intriguingly, those genes reflected a high percentage of known HCC or other cancer-relevant vital genes. These findings depicted activated pathways such as those for the cell cycle and DNA repair and repressed key metabolic pathways induced by aberrant DNA methylation of promoters and enhancers in HCC. Beyond those results, our perfectly matched methylome and transcriptome sequencing data from relatively large-scale paired tumoral and adjacent non-tumoral tissues also provide a valuable resource for follow-up studies in HCC, in which WGBS-based methylome data were insufficient.

## Data Availability Statement

The datasets presented in this study can be found in online repositories. The names of the repository/repositories and accession number(s) can be found at the NCBI SRA database [accession: PRJNA762641].

## Ethics Statement

The studies involving human participants were reviewed and approved by the Medical Ethics Committee of the First Affiliated Hospital of Zhejiang University. Written informed consent to participate in this study was provided by the participants’ legal guardian/next of kin.

## Author Contributions

Conceptualization, PH, MDL, and ZY. Data curation, PH, MX, HH, XZ, MDL, and ZY. Formal analysis, PH, HH, and XZ. Funding acquisition, MDL. Investigation, MX and HH. Methodology, PH, MX, XZ, and MDL. Project administration, MDL and ZY. Resources, MDL and ZY. Software, PH and XZ. Supervision, MDL and ZY. Validation, PH, MX, HH, and ZY. Visualization, PH. Writing—original draft, PH. Writing—review and editing, PH, MDL, and ZY. All authors contributed to the article and approved the submitted version.

## Funding

This study was supported in part by the China Precision Medicine Initiative (2016YFC0906300), Research Center for Air Pollution and Health of Zhejiang University, and the Independent Task of State Key Laboratory for Diagnosis and Treatment of Infectious Diseases.

## Conflict of Interest

The authors declare that the research was conducted in the absence of any commercial or financial relationships that could be construed as a potential conflict of interest.

## Publisher’s Note

All claims expressed in this article are solely those of the authors and do not necessarily represent those of their affiliated organizations, or those of the publisher, the editors and the reviewers. Any product that may be evaluated in this article, or claim that may be made by its manufacturer, is not guaranteed or endorsed by the publisher.
